# Reviewed article: Sousa EL, Rodrigues FC A, Saraiva JGC, Santos RSM, Zagury DGM, Brito GE. Geographic disparities and temporal trends regarding access to cancer treatment: a spatial analysis, Brazil, 2015-2022. Epidemiol Serv Saude. 2025:34;e20240725

**DOI:** 10.1590/S2237-96222026v35e20240725.a

**Published:** 2025-12-01

**Authors:** Maria Auxiliadora Parreiras Martins

**Affiliations:** 1 Universidade Federal de Minas Gerais, Belo Horizonte, MG, Brazil Belo Horizonte MG Brazil

## First round comments

Prezados autores(as), o manuscrito apresentado é interessante e seus achados úteis para melhor compreensão dos aglomerados espaciais e tendências temporais no deslocamento de pacientes para tratamento oncológico entre 2015 e 2022. Os aspectos apontados pelos revisores precisam ser seguidos para melhorar a qualidade do abstract, corpo do texto e profundidade da discussão. Seria importante comentar sobre possíveis fatores de confusão na observação dos achados. O período de 2020 a 2022 corresponde à pandemia de Covid-19 quando houve restrição de deslocamento para realização de tratamentos de saúde. Importante discutir seu impacto sobre os resultados. Após correções, o manuscrito poderá ser considerado para publicação.

Recommendation: Major revision

